# Breast cancer risk stratification for mammographic screening: A nation‐wide screening cohort of 24,431 women in Singapore

**DOI:** 10.1002/cam4.4297

**Published:** 2021-10-28

**Authors:** Peh Joo Ho, Fuh Yong Wong, Wen Yee Chay, Elaine Hsuen Lim, Zi Lin Lim, Kee Seng Chia, Mikael Hartman, Jingmei Li

**Affiliations:** ^1^ Genome Institute of Singapore Singapore Singapore; ^2^ Saw Swee Hock School of Public Health National University of Singapore and National University Health System Singapore Singapore; ^3^ Division of Radiation Oncology National Cancer Centre Singapore Singapore Singapore; ^4^ Division of Medical Oncology National Cancer Centre Singapore Singapore Singapore; ^5^ Department of Surgery Yong Loo Lin School of Medicine National University of Singapore Singapore Singapore

**Keywords:** breast cancer, Gail model, mammogram recall status, mammographic density, mammography screening

## Abstract

**Background:**

Breast cancer incidence is increasing in Asia. However, few women in Singapore attend routine mammography screening. We aim to identify women at high risk of breast cancer who will benefit most from regular screening using the Gail model and information from their first screen (recall status and mammographic density).

**Methods:**

In 24,431 Asian women (50–69 years) who attended screening between 1994 and 1997, 117 developed breast cancer within 5 years of screening. Cox proportional hazard models were used to study the associations between risk classifiers (Gail model 5‐year absolute risk, recall status, mammographic density), and breast cancer occurrence. The efficacy of risk stratification was evaluated by considering sensitivity, specificity, and the proportion of cancers identified.

**Results:**

Adjusting for information from first screen attenuated the hazard ratios (HR) associated with 5‐year absolute risk (continuous, unadjusted HR [95% confidence interval]: 2.3 [1.8–3.1], adjusted HR: 1.9 [1.4–2.6]), but improved the discriminatory ability of the model (unadjusted AUC: 0.615 [0.559–0.670], adjusted AUC: 0.703 [0.653–0.753]). The sensitivity and specificity of the adjusted model were 0.709 and 0.622, respectively. Thirty‐eight percent of all breast cancers were detected in 12% of the study population considered high risk (top five percentile of the Gail model 5‐year absolute risk [absolute risk ≥1.43%], were recalled, and/or mammographic density ≥50%).

**Conclusion:**

The Gail model is able to stratify women based on their individual breast cancer risk in this population. Including information from the first screen can improve prediction in the 5 years after screening. Risk stratification has the potential to pick up more cancers.

## Graphical abstract

Readily available information from the first mammography screening should be used to help women make informed choices on attending regular screening. Having a false‐positive result or dense breast should not deter from mammography screening; conversely, they should consciously attend mammography screening.

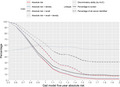

## 1 INTRODUCTION

The number of new breast cancer cases diagnosed has been on a rapid rise in Asia.^1^, ^2^, ^3^ The burden of the disease is notable in Singapore, with a 17% increase in the age‐standardized incidence rate within one decade (70.8 per 100,000 in the period 2012–2017, compared to 60.3 per 100,000 in 2003–2007) (National Registry of Diseases 4). In addition, the 5‐year survival rate in Singapore (80% in 2010–2014) is lower than the rates of other countries in Asia, such as China (83%), Japan (89%) and South Korea (87%).

Breast tumors are most treatable when small and discovered early. Early detection of less‐advanced breast cancers requires less aggressive treatment and confers a survival benefit.^5^ Mammography is the current standard used in population‐based breast cancer screening. A substantial reduction in late‐stage disease and breast cancer mortality is credited to screening mammography.^5^, ^6^

Mammography screening is not perfect. Despite advances in mammography techniques, it is estimated that 10%–29% of breast cancers are missed.^7^, ^8^ False‐positive findings, overdiagnosis, and overtreatment of small tumors that are unlikely to progress to fatal disease are other often‐cited negative outcomes of mammography screening programs.^9^ A risk‐stratified screening program, where women at high risk of the disease are strongly recommended for regular screening, may improve the current program in favor of finding more treatable cancers and make screening programs more effective.^10^

Traditional breast cancer risk factors considered for risk‐based screening include family history of the disease, ethnicity, age, breast biopsy results, and breast density.^11^ A widely used tool, the Gail model, also known in clinical practice as the Breast Cancer Risk Assessment Tool (BCRAT), is a prediction tool that estimates a woman's risk of developing breast cancer over time.^12^ It incorporates personal details on family history of breast cancer, as well as medical and reproductive history. The tool is originally developed and validated for White females with no history of in situ or invasive breast cancer. For use in Asian populations, the accuracy of the Gail model remains debatable.^13^ In addition, information derived from mammography screening visits can improve individual breast cancer risk prediction. For example, women with higher breast density are at higher risk of developing breast cancer in the subsequent years from screening.^14^, ^15^, ^16^ False‐positive results are also associated with increased breast cancer risk for over a decade.^17^

The campaign for nation‐wide breast cancer screening in Singapore started in 2002. Under the program, biennial mammography screening is heavily subsidized for citizens and permanent residents above the age of 50 years.^18^ While 66% of the target population reported attending at least one screen, compliance to routine screening is an issue.^18^ In the period 2001–2010, the majority (60%–65%) of women aged 50–69 attending screening were there for their first mammogram.^18^ However, the percentage of women reported to return for a second mammogram in the same period was only 26.4% (2003), 34.2% (2005) and 12.6% (2007).^18^ In recent years, among women who attended their first screen, less than one in five went back for the subsequent screening after 2 years (13.6% in 2016, 18.6% in 2017, and 18.4% in 2018) (Health Promotion Board, Singapore).

A local focus group study identified concerns such as painful and uncomfortable experience, low perceived self‐risk of breast cancer, need for timely reminders, and misinformation as barriers to routine screening.^19^ Many studies have found that a personalized approach, in the form of tailored invites and reminders, is superior over usual care in terms of effectiveness and cost.^20^, ^21^ The involvement of primary care physicians or healthcare personnel in making informed decisions is also found to increase screening uptake and subsequent visits.^22^, ^23^ A paradigm shift to risk‐based screening and tailored interventions thus has the potential to improve the attendance and adherence to the program.

In this population‐based study comprising 24,431 women attending mammography screening in Singapore, we attempt to identify women who would benefit most from regular screening, using the Gail model and additional information on false‐positive results and breast density from the first screen.

## 2 METHODS

### 2.1 Study population

The Singapore Breast Screening Project (SBSP) was designed as a population‐based randomized trial on mammography screening of women aged 50–64 years, with 69,473 (41.7%) women randomly selected and invited for this single free screening.^24^ Of those invited, women were excluded if they had previous cancers, except non‐melanoma skin cancers; have had mammograms within the past year or a biopsy within 6 months (these women would be on active follow‐up and thus not be suitable for screening mammography); or were pregnant.^24^ Screening centers were set‐up at the Singapore General hospital and Toa Payoh Hospitals.^24^ Of the 28,235 who participated between October 1994 and February 1997, 24,553 women had available and viable mammograms for mammographic density reading.^25^, ^26^

Breast cancer cases were identified via linkage with the Singapore Cancer Registry with the latest date of occurrence set at December 2007. We excluded 98 women whose breast cancer was detected during screening, 18 whose self‐reported age at first birth was before their age of menarche, three with invalid age and two with unknown date of diagnosis. A total of 24,431 women were included. In this dataset, 117 breast cancers were diagnosed within 5 years after screening; 21 were diagnosed between 12 and 24 months from screening This study was approved by the SingHealth Centralised Institutional Review Board (IRB: 2009/985/B).

Details on self‐reported breast cancer risk factors included in the Gail model—age at menarche, age at first live birth, prior biopsy, and family history of breast cancer—were obtained from the questionnaire administered at recruitment. Women who had a biopsy done during screening were coded as 1 for the risk factor biopsy and the rest were coded as 0 (no biopsy done in the past year). Family history of breast cancer was ascertained as no/yes for mother, sister(s), and daughter(s). Number of first‐degree relatives with breast cancer was coded as 0 (no family history), 1 (either mother, sister, or daughter), or 2 (any two first‐degree relatives). Information on recall for additional follow‐up due to suspicious findings on the mammogram (recall [no/yes]) and percent mammographic density was obtained from the first screening result mediolateral oblique views of both breasts were used in measuring mammographic density, detailed process is in Lee et al..^26^ Mammographic density was categorized as – <15%, 15%–25%, 25%–50%, and ≥50%, where the lower bound was included but the upper bound was not for the second and third category. Body mass index (kg/m^2^, BMI) was retrieved from the questionnaire, and missing values were replaced by the mean. Missing values for the Gail model variables were coded as the reference level.^12^

### 2.2 Statistical analysis

Breast cancer risk factors, recall status, and mammographic density were compared between 117 women who develop breast cancer within 5 years from screening date and 24,314 women who did not (Fisher's exact test for categorical variables and Kruskal–Wallis test for continuous variables).

To obtain the 5‐year absolute risk of each individual, we first estimated the Gail model relative risk score using Asian weights and attributable risks from the BCRA package in R.^12^ Modifications of the “recode.check”, “relative.risk,” and “absolute.risk” function allowed for (1) the major ethnicities (Chinese, Malay and Indian) to be used in place of those provided, and (2) the use of breast cancer incidence rates and mortality rates of Singapore (Supplementary Methods). Breast cancer incidence rates in the period 2013–2017, from the Singapore Cancer Registry, and age‐specific mortality rates in 2016, from the Department of Statistics (Singapore) were used in the estimation of absolute risk (27, Department of 28).

The association of the Gail model 5‐year absolute risk, recall status, and mammographic density with breast cancer development within 5 years from screening date was studied using the Cox proportional hazards model. In addition, we adjusted for body mass index, which is a well‐known confounder of the mammographic density and breast cancer risk association.^29^

Breast cancer case/non‐case classification was done using logistic models with one or more factors (Gail model 5‐year absolute risk, recall status, percent mammographic density). The discriminatory ability of the logistic model was measured using the area under the receiver operating characteristic curve (AUC). Sensitivity and specificity reported were based on cut‐off points identified by Youden's J statistic.

The performances of the Gail model 5‐year absolute risk, recall status, and mammographic density as risk stratification tools were evaluated by considering sensitivity, specificity and the proportion of all breast cancers identified.^30^ To evaluate the added value of the risk predictors studied (mammographic density and recall status) to an age‐based screening paradigm, a hypothetical comparison was made by simulating a random sample of the same percentage of women screened. The top percentile category was compared to the remaining to assess the potential for a stratified breast cancer screening.

All analyses were performed in R version 4.0.2.

## 3 RESULTS

A total of 24,431 women were included, with 117 women developing breast cancer within 5 years of their screening date. Age at study entry was not significantly different between women who developed breast cancer and those who did not (Table 1). Women who developed breast cancer were more likely to be of older age at first live birth or nulliparous (*p* < 0.001), to have a family history of breast cancer (*p* < 0.001), to be recalled for additional follow‐up (*p* < 0.001), or to have higher breast density (*p* < 0.001) as compared with women who did not.

**TABLE 1:** Description of study population

	Breast cancer developed within 5 years post‐diagnosis		*p*‐value
	No (*n* = 24,314)	Yes (*n* = 117)	
Median age at study entry (IQR)	57 (54–61)	57 (55–60)	0.414
Ethnicity			
Chinese	20,705 (85.2)	105 (89.7)	0.345
Malay	1305 (5.4)	2 (1.7)	
Indian	1123 (4.6)	5 (4.3)	
Others	1181 (4.9)	5 (4.3)	
Age at menarche (years)			
≥14	15,827 (65.1)	68 (58.1)	0.115
12–13	7698 (31.7)	42 (35.9)	
<12	777 (3.2)	7 (6.0)	
Missing	12 (0.0)	0 (0.0)	
Age at first live birth (years)			
<20	4118 (16.9)	16 (13.7)	<0.001
20–24	9136 (37.6)	27 (23.1)	
25–29 or nulliparous	8176 (33.6)	49 (41.9)	
≥30	2731 (11.2)	24 (20.5)	
Missing	153 (0.6)	1 (0.9)	
Ever had a biopsy			
No	24,103 (99.1)	115 (98.3)	0.272
Yes	211 (0.9)	2 (1.7)	
Family history (first‐degree) of breast cancer			
0	23,703 (97.5)	106 (90.6)	<0.001
1 (mother, sister, or daughter)	600 (2.5)	11 (9.4)	
2 (any two first‐degree relatives)	11 (0.0)	0 (0.0)	
Mammogram recall status			
No	22,609 (93.0)	87 (74.4)	<0.001
Yes	1,705 (7.0)	30 (25.6)	
Median mammographic density (IQR)	18.29 (12.32–26.09)	24.19 (16.60–31.76)	<0.001
Mammographic density (categorical)			
0–15%	8,944 (36.8)	21 (17.9)	<0.001
15–25%	8,586 (35.3)	44 (37.6)	
25–50%	6,514 (26.8)	50 (42.7)	
≥50%	270 (1.1)	2 (1.7)	
Median body mass index, kg/m^2^ (IQR)	24.28 (21.88–26.89)	24.59 (22.57–26.91)	0.432
Missing	7	0	
Median Gail model relative risk (IQR)	1.42 (1.32–1.87)	1.74 (1.32–2.29)	<0.001
Median 5‐year absolute risk (IQR)	0.85 (0.72–1.06)	0.99 (0.80–1.28)	<0.001

Abbreviation: IQR, interquartile range.

### 3.1 Distribution of Gail model relative risk and 5‐year absolute risk

**FIGURE 1:**
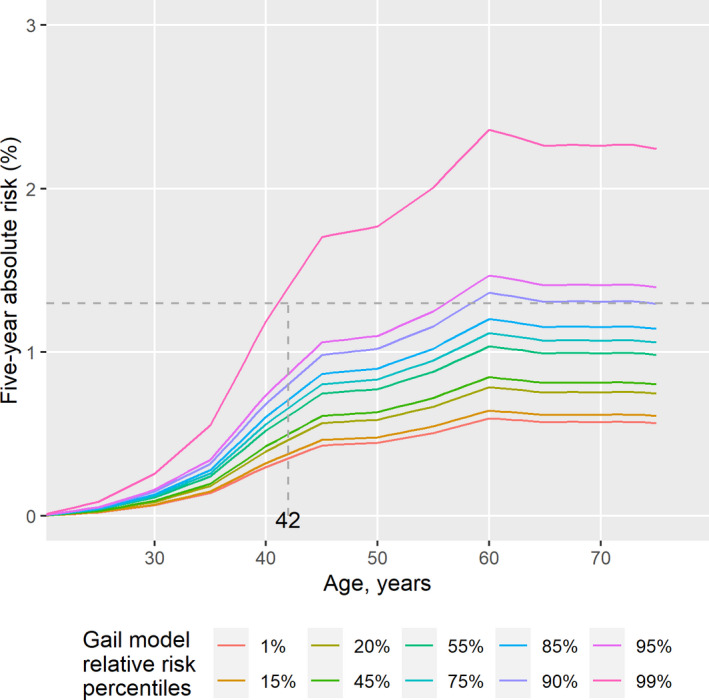
Gail model 5‐year absolute risk of developing breast cancer. Percentiles are obtained from the distribution of relative risks in all women at the age of enrolment. The relative risks are as estimated from BCRA package in R, using weights and attributable risk of Asians. Absolute risk were estimated using breast cancer incidence rates from the Singapore Cancer Registry and mortality rates obtained from the Department of Statistics, Singapore. The relative risk of each percentile is presented in Table S1

The distributions of the Gail model relative risk and 5‐year absolute risk were skewed, with a median of 1.4 (range: 1.0–12.0) for relative risk and a median of 0.9% (range: 0.5%–6.3%) for absolute risk (Figure S1). Women at the top one percentile of the Gail model relative risk were of ~4‐fold higher risk of developing breast cancer than those of the bottom one percentile (Table S1). The projected 5‐year absolute risk reached 1.3% (i.e., the 5‐year risk of an average Caucasian woman at age 50 years^31^) at the age of 42 years in women with a relative risk in the top 1% of this population (Figure 1).

### 3.2 Information from first screen

All women diagnosed with breast cancer at first screen were excluded from our analysis. Women who were recalled for follow‐up were at a higher risk of developing breast cancer in the next 5 years (HR [95% CI]: 4.5 [3.0–6.9], *p* < 0.001) (Table 2). This increased risk persisted after adjusting for the Gail model 5‐year absolute risk and mammographic density (HR [95% CI]: 3.6 [2.3–5.5], *p* < 0.001). Having a higher mammographic density was significantly associated with breast cancer occurrence (adjusted HR_[15%–25%] vs_. _<15%_ [95% CI]: 1.9 [1.1–3.2], *p* = 0.022, HR_[25%–50%])vs. <15%_ [95% CI]: 2.6 [1.5–4.3], *p* < 0.001), and HR_[≥50%]) vs. <15%_ [95% CI]: 2.4 [0.6–10.3], *p* = 0.234), adjusted for the Gail model 5‐year absolute risk and recall status (Table 2). Adding continuous BMI to the model with Gail model 5‐year absolute risk, recall status, and mammographic density, increased the model's discriminatory ability (AUC increased from 0.703 [0.653–0.753] to 0.722 [0.671–0.772]). The p‐value for BMI in the model was 0.053. The observed HRs for other variables were appreciably unchanged.

**TABLE 2:** Association between 5‐year absolute risk category (as estimated from the Gail model) and risk of breast cancer within 5 years of screening date, in Singapore Breast Screening Project (SBSP)

	Unadjusted		Adjusted*	
	HR (95% CI)	*p*‐value	HR (95% CI)	*p*‐value
Gail model 5‐year absolute risk				
Continuous	2.3 (1.8–3.1)	<0.001	1.9 (1.4–2.6)	<0.001
Mammogram recall status				
No	1.0 (Reference)		1.0 (Reference)	
Yes	4.5 (3.0–6.9)	<0.001	3.6 (2.3–5.5)	<0.001
Mammographic density				
<15	1.0 (Reference)		1.0 (Reference)	
15 to 25	2.2 (1.3–3.7)	0.003	1.9 (1.1–3.2)	0.014
25 to 50	3.3 (2.0–5.4)	<0.001	2.6 (1.5–4.3)	<0.001
≥50	3.1 (0.7–13.4)	0.121	2.4 (0.6–10.3)	0.234

* Adjusted model has recalled mammogram status and 5‐year absolute risk percentile category in the model. ^With the exception of the first and last categories, the lower bound was included but the upper bound was not.

### 3.3 Binary classification of risk status

Ten percent of the women had 1.3% and above the Gail model 5‐year absolute risk at enrolment. Of these 1.2% (*n* = 28) developed breast cancer in the next 5 years; these breast cancer cases formed 24% (*n* = 28, of 117) of all breast cancer occurrence in that period. Twenty‐five percent (*n* = 30 of 117) of all breast cancer occurrence were from women who were recalled at first screen, and 2% (*n* = 2 of 117) had mammographic density above 50%. Forty‐four percent of all breast cancer occurrence can be identified when we defined high risk as a combination of having any one of the following, (i) the Gail model 5‐year absolute risk of 1.3% and above, (ii) positive recall status, or (iii) mammographic density of 50% and above. This was observed due to the low concordance between the three criteria (Kappa ranged from 0.005 to 0.072, Table S2).

### 3.4 Model performance

**FIGURE 2:**
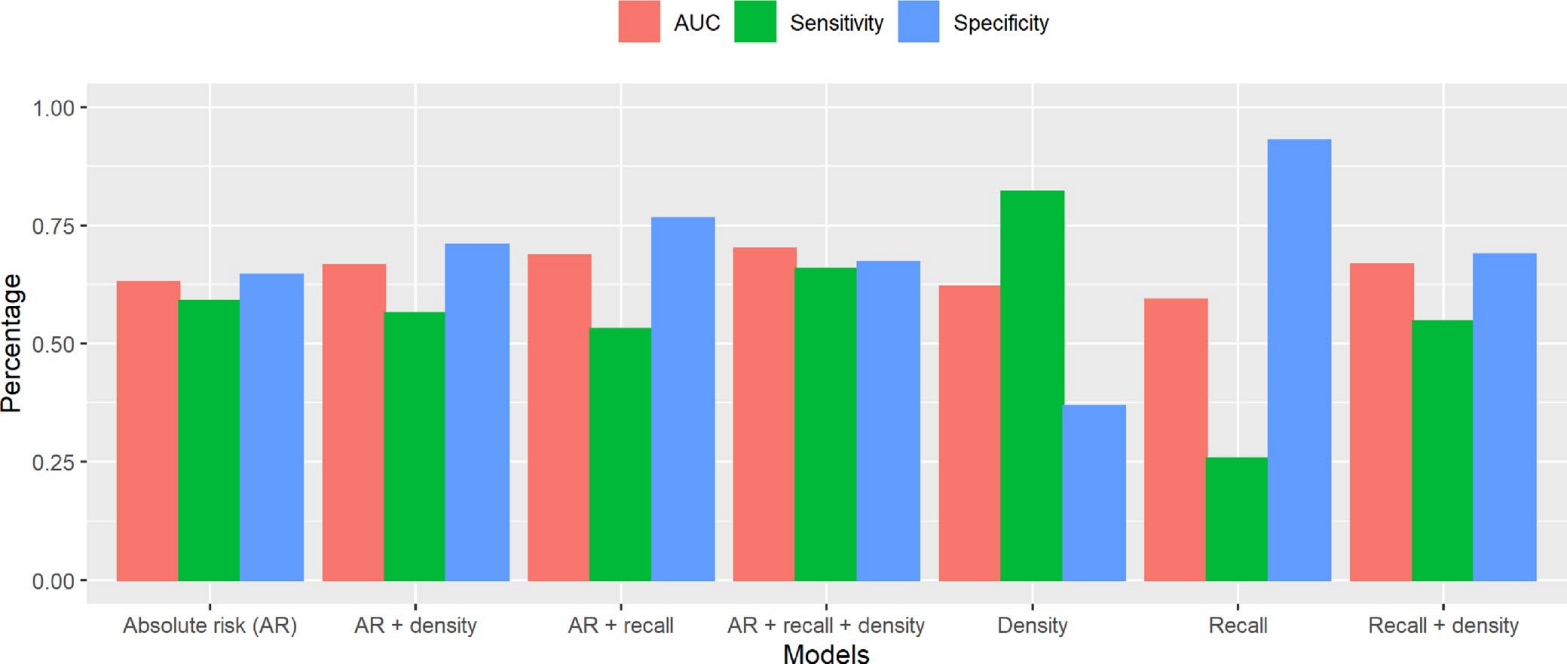
Sensitivity and specificity for predicting breast cancer occurrence in the subsequent five years by one or more factors (Gail model 5‐year absolute risk (continuous, AR), mammogram recall (binary, recall) and percent density (categorical, <15, 15–25, 25–50, and ≥50) using the logistic regression model

The Gail model 5‐year absolute risk had a discriminatory ability of 0.615 (95% CI: 0.559–0.670) (Table S3). An improvement in discriminatory ability was observed when information from the first screen (recall status and mammographic density) was added (AUC [95% CI]: 0.703 [0.653–0.753]). The best‐performing model based on AIC was the model with the Gail model 5‐year absolute risk, recall status, and mammographic density (AIC = 1427). While the discriminatory abilities of the models may be close (AUC ranges from 0.593 to 0.703), sensitivity and specificity were different based on cut‐offs identified by maximizing the Youden's J statistic, (Figure 2 and Table S3). For example, recall status has low sensitivity (0.256) and high specificity (0.930). In contrast, mammographic density has a high sensitivity (0.821) and a low specificity (0.368).

### 3.5 Performance of stratified risk management as compared to a random sample

**FIGURE 3:**
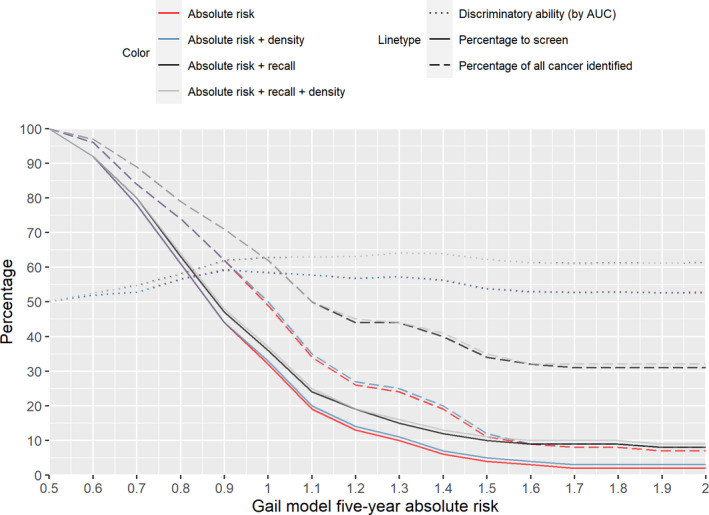
The proportion of women to screen and the proportion of all breast cancer identified by the combination of binary variables (absolute risk, recall, density) at different thresholds of Gail model 5‐year absolute risk. Discriminatory ability, of the combination of binary variables, is measured by the area under the receiver operating curve (AUC) using prediction from a logistic regression model (dotted lines). Solid lines denote the proportion of women considered as high risk, hence also the proportion of women to be screened (red, based on Gail 5‐year absolute risk≥threshold (x‐axis) only; blue, combination of Gail and mammographic density ≥50%; black, Gail and density and recall status (yes/no); grey, combination of Gail, density, and recall status). Dashed lines denote the percentage of all cancer identified in each corresponding group. The addition of recall status in risk classification resulted in a higher proportion of cancers identified (larger gap between dotted lines) compared to the difference in number of women needed to be screened (gap between solid lines)

Based on a binary division of breast cancer risk (high or low) by the different scoring systems (the Gail model 5‐year absolute risk, combinations with recall status and mammographic density), screening only the high‐risk group out‐performs screening a random sample of a similar size in terms of the number of breast cancers detected (Figure 3 and Table S4). Thirty‐five percent of all breast cancer cases were detected in 20% of the women with the highest Gail model absolute risk. In contrast, 38% of all breast cancers diagnosed in the next 5 years were detected in only 12% of the women with the highest risk based on 5‐year absolute risk (top 5 percentile) and mammogram recall status group (Table S4). In spite of a smaller proportion of women screened, a larger gain in the proportion of breast cancers identified was achieved by screening women with any of the high‐risk criteria (the Gail model 5‐year absolute risk above 1.2%, recalled mammogram or breast density ≥50%) (Figure 3).

## 4 DISCUSSION

As the most common cancer diagnosed among women with tangible benefits from early detection, breast cancer is an ideal disease for an organized population cancer screening program.^32^ A successful screening program is largely based on continuous monitoring. However, getting women to attend regular screening is challenging.^32^, ^33^ Even when cost or convenience are taken out of the concerns, experiential and psychological obstacles often lead to the underutilization of mammography services in many countries, including Singapore.^34^

Our results showed that risk stratification of screening participants using breast cancer risk factors included in the Gail model picked up more cancers than a random sample among those in the highest risk group. In addition, information from a single mammogram, such as mammographic density and recall status, helped to identify women at higher risk of developing breast cancer. Each risk model has its own limits and flaws in terms of sensitivity and specificity. The best performing discriminatory ability (AUC = 70%) was observed when the Gail model 5‐year absolute risk, mammographic density, and recall status were considered together in our study, making it possible to generate individual breast cancer risk profiles at first screen. The tailored information may be communicated to the screening participant to raise breast cancer awareness and education. Women at high risk may be particularly targeted to attend regular screening.^35^ Empowered with knowledge, women may develop a personal stake in preventing the disease and find it more motivating to attend subsequent mammograms. Nonetheless, the acceptability of personalized risk‐based breast cancer screening and prevention needs to be assessed in the local population.^36^

For a mammography screening program to be effective in catching breast cancers early, the screening interval (i.e. time between two screening mammograms) must be shorter than the time the tumor takes to grow larger than the preclinical screen‐detectable phase, otherwise known as “sojourn time”.^37^, ^38^ There is considerable homogeneity in breast cancer screening guidelines across different countries for women aged 50 to 69, for whom biennial screening is recommended. The United Kingdom is an exception; a longer screening interval of 3 years is recommended.^39^ Recommendations for younger women between the ages of 40–49 are less consistent. In most countries, there are no screening guidelines for this age group. In Sweden, younger women are recommended to go for biennial mammograms like their older counterparts.^39^ In the United States and in Singapore, an annual mammogram is recommended selectively.^39^

At the individual level, the start age and screening interval may be earlier and shorter for women at higher risk of breast cancer.^40^ Women with a high breast cancer risk profile may be at the same risk as a 50‐year‐old woman at a younger age. A breast cancer risk assessment and a baseline mammogram at age 40 has the potential to aid women in making informed decisions on whether to go for a mammogram every year until age 49 before following standard recommendations to do biennial screening.

Our results show that high‐risk individuals, especially those with a high Gail model 5‐year absolute risk or who were recalled previously, are more likely to benefit from adherence to routine mammography. The accuracy of mammography is lower in women with dense breasts. While the sensitivity of the method is approximately 77%–95% across all ages, it can be as low as 63% in women with high mammographic density.^41^, ^42^ Cancers may be missed due to a masking effect hence, a shorter screening interval may be of advantage. However, false‐positive mammograms that lead to unnecessary invasive biopsies are also more common for dense breasts, a consideration that must be noted. The current mammography screening paradigm stands to benefit from using personalized risk profiles for targeted intervention and reminders to attend routine screening.

It should be noted that the incidence and mortality rates used in the calculation of the Gail model in this study are from more recent years as compared to the period of data collection. Sensitivity analysis using incidence rates (period: 1993–1997) and mortality rates (year 1997) closer to the recruitment period showed a shift in distribution of 5‐year absolute risk to that of lower risk (median in the period 1993–1997 [range]: 0.5 [0.2–1.2]; vs. period 2013–2017: 0.9 [0.5–6.3]), as expected of a period of lower breast cancer incidence. Nevertheless, the result from the sensitivity analysis shows that the majority of breast cancer cases that occurred within 5 years of screening can be identified in the top five percentile of the 5‐year absolute risk and/or positive mammogram recall status group (i.e., 34% of all breast cancer cases were identified in the high‐risk group, which comprises of 11% of all women). In addition, the relative associations between the 5‐year absolute risk and breast cancer occurrence remain (adjusted HR in the period 1993–1997 [95% CI]: 2.8 [1.7–4.5]); vs. period 2013–2017: 1.9 [1.4–2.6]).

The strength of our study is that it is a nation‐wide screening program with over 24,000 participants from an unselected population of women aged 50–64 years.^25^, ^26^ Mammogram screening was provided at no charge to the participants, thus eliminating cost as a barrier. The coverage of the national cancer registry in Singapore is excellent, with completeness estimated to be ~98%.^43^ Leaving Singapore is the main reason to be lost to follow‐up, given that the women in our study are the Pioneer generation few left Singapore–we did not observe any loss to follow‐up.

Several limitations apply to our study. SBSP was established over two decades ago as a pilot study to support a nation‐wide mammography screening service in Singapore.^5^ As a result, potential sources of bias for the investigation of our current study aims cannot be addressed retrospectively. The age‐standardized incidence rate (per 100,000 annually) of the disease has increased from ~40 since then to ~65 currently. Although the incidence rate may not be an accurate reflection of today's statistics, it highlights a growing importance to improve the current screening paradigm. In recent years, less than one in five women attended subsequent screening after 2 years their first (18.4% in 2018) (Health Promotion Board, Singapore). The low response rate (41%) in the initial randomized trial may result in a response bias where we have more health conscious women attending the screening trial; the reasons for non‐response were not recorded. Hence, SBSP may not fully represent the general screening population in Singapore. However, the main objective of this study was to understand the relative effects of the Gail model risk estimates and information at first screen and breast cancer risk, which is not enhanced by representativeness.^44^ In addition, major demographic characteristics, such as ethnic group distributions, are similar to that of the general population (Government's Population‐in‐Brief report). Chay et al. assessed the validity of the Gail model on SBSP and reported that the model overestimates the individual risk for breast cancer in Singapore.^45^ However, a conservative approach to identifying high‐risk individuals in a risk‐based screening program may be more effective in detecting more cancers. Nonetheless, future work using appropriately calibrated models will be needed for better prediction of risk. The Gail model, recall status and mammographic density are only a few of the risk indicators that can be included in a risk‐based screening program. Genetic risk factors, such as carriership of *BRCA*1/2 genes or polygenic risk scores, will help further identify high‐risk individuals. The Women Informed to Screen Depending on Measures of Risk (WISDOM) trial, for example, incorporated genetic risk (single nucleotide polymorphisms and *BRCA1*/*2*, *TP53*, *PTEN*, *STK11*, *CDH1*, *ATM*, *PALB2*, or *CHEK2* mutation carriers) in their assessments.^31^, ^46^ Biospecimens were not collected from the participants in this study. Due to the limited number of breast cancer cases, we were unable to perform subgroup and interaction analyses.

## 5 CONCLUSION

The Gail model is able to stratify women based on their individual breast cancer risk. Information from the first screen improved the prediction for cancers developing in the next 5 years after screening. However, challenges in communicating individual breast cancer risk and other barriers to routine mammography screening (cultural or psychological) will need to be overcome to increase screening uptake and adherence.

## CONFLICT OF INTEREST

None declared.

## AUTHOR CONTRIBUTIONS

Study design: PJH, JL, and FYW; Provision of data: FYW, WYC, and EHL; Statistical analysis: PJH and JL; Writing of the first draft: PJH and JL; Clinical expertise: FYW, WYC, EHL, and MH; Epidemiological expertise: MH and CKS. All authors read and approved the final version of the paper.

## Supporting information

Supplementary Material

Supplementary Material

## Data Availability

The datasets used and/or analyzed during the current study are available from Dr. Wong Fuh Yong (wong.fuh.yong@singhealth.com.sg) upon reasonable request.
