# Supplementary material for: Breast cancer risk stratification for mammographic screening: A nation‐wide screening cohort of 24,431 women in Singapore
Source: Cancer Med. 2021 Oct 28;10(22):8182–91. doi: 10.1002/cam4.4297 (PMC8607242; doi:10.1002/cam4.4297)
Supplement: Supplementary file 1 — Supplementary Material [file CAM4-10-8182-s002.docx]

**Breast cancer risk stratification for mammographic screening:
A nation-wide screening cohort of 24,431 women in Singapore**

**Supplementary Method**

The following code shows the edits done to the BCRA package from R. It requires:

1. The BCRA library (Gail et al., 1989)
2. The incidence and mortality rates of Singapore, provided as a separate file (Singapore breast cancer incidence and mortality rates.csv)

Breast cancer incidence rates in the period 2013-2017, from the Singapore Cancer Registry, and age-specific mortality rates in 2016, from the Department of Statistics (Singapore) were used in the estimation of absolute risk (National Registry of Diseases Office, 2017; Department of Statistics, 2017). Incidence rates are ethnicity specific while mortality rates are based on all females.

Modified lines and regions are indicated by “EDITED”

lambda <- read.csv("Singapore breast cancer incidence and mortality rates.csv")

library(BCRA)

recode.check <- function (data, Raw_Ind = 1){

Error_Ind <- rep(0, dim(data)[1])

data$T1 <- as.numeric(as.character(data$T1)) ## EDITED

data$T2 <- as.numeric(as.character(data$T2)) ## EDITED

set_T1_missing <- data$T1

set_T2_missing <- data$T2

set_T1_missing[which((data$T1 < 20 | data$T1 >= 90) | data$T1 >=

data$T2)] <- NA

set_T2_missing[which(data$T2 > 90 | data$T1 >= data$T2)] <- NA

Error_Ind[is.na(set_T1_missing)] <- 1

Error_Ind[is.na(set_T2_missing)] <- 1

if (Raw_Ind == 1) {

NB_Cat <- rep(-1, dim(data)[1])

NB_Cat[which((data$N_Biop == 0 | data$N_Biop == 99) &

data$HypPlas != 99)] <- "A"

Error_Ind[which(NB_Cat == "A")] <- 1

NB_Cat[which((data$N_Biop > 0 & data$N_Biop < 99) & (data$HypPlas !=

0 & data$HypPlas != 1 & data$HypPlas != 99))] <- "B"

Error_Ind[which(NB_Cat == "B")] <- 1

NB_Cat[which(NB_Cat == -1 & (data$N_Biop == 0 | data$N_Biop ==

99))] <- 0

NB_Cat[which(NB_Cat == -1 & data$N_Biop == 1)] <- 1

NB_Cat[which(NB_Cat == -1 & (data$N_Biop >= 2 | data$N_Biop !=

99))] <- 2

NB_Cat[which(NB_Cat == -1)] <- NA

AM_Cat <- rep(NA, dim(data)[1])

AM_Cat[which((data$AgeMen >= 14 & data$AgeMen <= data$T1) |

data$AgeMen == 99)] <- 0

AM_Cat[which(data$AgeMen >= 12 & data$AgeMen < 14)] <- 1

AM_Cat[which(data$AgeMen > 0 & data$AgeMen < 12)] <- 2

AM_Cat[which(data$AgeMen > data$T1 & data$AgeMen != 99)] <- NA

AM_Cat[which(data$Race == 2 & AM_Cat == 2)] <- 1

AF_Cat <- rep(NA, dim(data)[1])

AF_Cat[which(data$Age1st < 20 | data$Age1st == 99)] <- 0

AF_Cat[which(data$Age1st >= 20 & data$Age1st < 25)] <- 1

AF_Cat[which((data$Age1st >= 25 & data$Age1st < 30) |

data$Age1st == 98)] <- 2

AF_Cat[which(data$Age1st >= 30 & data$Age1st < 98)] <- 3

AF_Cat[which(data$Age1st < data$AgeMen & data$AgeMen !=

99)] <- NA

AF_Cat[which(data$Age1st > data$T1 & data$Age1st < 98)] <- NA

AF_Cat[which(data$Race == 2)] <- 0

NR_Cat <- rep(NA, dim(data)[1])

NR_Cat[which(data$N_Rels == 0 | data$N_Rels == 99)] <- 0

NR_Cat[which(data$N_Rels == 1)] <- 1

NR_Cat[which(data$N_Rels >= 2 & data$N_Rels < 99)] <- 2

NR_Cat[which((data$Race >= 6 & data$Race <= 11) & NR_Cat ==

2)] <- 1

}

if (Raw_Ind == 0) {

NB_Cat <- data$N_Biop

AM_Cat <- data$AgeMen

AF_Cat <- data$Age1st

NR_Cat <- data$N_Rels

}

R_Hyp <- rep(NA, dim(data)[1])

R_Hyp[which(NB_Cat == 0)] <- 1

R_Hyp[which((NB_Cat != "A" & NB_Cat > 0) & data$HypPlas ==

0)] <- 0.93

R_Hyp[which((NB_Cat != "A" & NB_Cat > 0) & data$HypPlas ==

1)] <- 1.82

R_Hyp[which((NB_Cat != "A" & NB_Cat > 0) & data$HypPlas ==

99)] <- 1

set_HyperP_missing <- data$HypPlas

set_R_Hyp_missing <- R_Hyp

set_HyperP_missing[which(NB_Cat == "A")] <- "A"

set_R_Hyp_missing[which(NB_Cat == "A")] <- "A"

set_HyperP_missing[which(NB_Cat == "B")] <- "B"

set_R_Hyp_missing[which(NB_Cat == "B")] <- "B"

set_Race_missing <- data$Race

Race_range <- seq(1, 15) ## EDITED

set_Race_missing[-which(data$Race %in% Race_range)] <- "U"

Error_Ind[which(is.na(NB_Cat) | is.na(AM_Cat) | is.na(AF_Cat) |

is.na(NR_Cat) | set_Race_missing == "U")] <- 1

AF_Cat[which(data$Race == 2)] <- 0

AM_Cat[which(data$Race == 2 & AM_Cat == 2)] <- 1

NB_Cat[which((data$Race %in% c(3, 5)) & (data$N_Biop %in%

c(0, 99)))] <- 0

NB_Cat[which((data$Race %in% c(3, 5)) & NB_Cat == 2)] <- 1

AM_Cat[which(data$Race == 3)] <- 0

AF_Cat[which((data$Race %in% c(3, 5)) & (data$Age1st != 98) &

AF_Cat == 2)] <- 1

AF_Cat[which((data$Race %in% c(3, 5)) & AF_Cat == 3)] <- 2

NR_Cat[which((data$Race %in% c(3, 5)) & NR_Cat == 2)] <- 1

NR_Cat[which((data$Race >= 6 & data$Race <= 11) & NR_Cat ==

2)] <- 1

CharRace <- rep(NA, dim(data)[1])

CharRace[which(data$Race == 1)] <- "Wh"

CharRace[which(data$Race == 2)] <- "AA"

CharRace[which(data$Race == 3)] <- "HU"

CharRace[which(data$Race == 4)] <- "NA"

CharRace[which(data$Race == 5)] <- "HF"

CharRace[which(data$Race == 6)] <- "Ch"

CharRace[which(data$Race == 7)] <- "Ja"

CharRace[which(data$Race == 8)] <- "Fi"

CharRace[which(data$Race == 9)] <- "Hw"

CharRace[which(data$Race == 10)] <- "oP"

CharRace[which(data$Race == 11)] <- "oA"

CharRace[which(data$Race == 12)] <- "SG" ## EDITED

CharRace[which(data$Race == 13)] <- "CN" ## EDITED

CharRace[which(data$Race == 14)] <- "MY" ## EDITED

CharRace[which(data$Race == 15)] <- "IN" ## EDITED

CharRace[which(is.na(CharRace))] <- "??"

recode_check <- cbind(Error_Ind, set_T1_missing, set_T2_missing,

NB_Cat, AM_Cat, AF_Cat, NR_Cat, R_Hyp, set_HyperP_missing,

set_R_Hyp_missing, set_Race_missing, CharRace)

recode_check <- data.frame(recode_check, row.names = NULL)

return(recode_check)

}

relative.risk <- function (data, Raw_Ind = 1){

White_Beta <- c(0.5292641686, 0.0940103059, 0.2186262218,

0.9583027845, -0.288042483, -0.1908113865)

Black_Beta <- c(0.1822121131, 0.2672530336, 0, 0.4757242578,

-0.1119411682, 0)

Hspnc_Beta <- c(0.0970783641, 0, 0.2318368334, 0.166685441,

0, 0)

FHspnc_Beta <- c(0.4798624017, 0.2593922322, 0.4669246218,

0.9076679727, 0, 0)

Other_Beta <- c(0.5292641686, 0.0940103059, 0.2186262218,

0.9583027845, -0.288042483, -0.1908113865)

Asian_Beta <- c(0.55263612260619, 0.07499257592975, 0.27638268294593,

0.79185633720481, 0, 0)

Wrk_Beta_all <- rbind(White_Beta, Black_Beta, Hspnc_Beta,

Other_Beta, FHspnc_Beta, Asian_Beta, Asian_Beta, Asian_Beta,

Asian_Beta, Asian_Beta, Asian_Beta,

Asian_Beta, Asian_Beta, Asian_Beta, Asian_Beta) ## EDITED

LP1 <- rep(NA, dim(data)[1])

LP2 <- rep(NA, dim(data)[1])

check_cov <- recode.check(data, Raw_Ind)

NB_Cat <- check_cov$NB_Cat

NB_Cat[which(NB_Cat == "A" | NB_Cat == "B")] <- NA

NB_Cat <- as.numeric(as.character(NB_Cat))

AM_Cat <- as.numeric(as.character(check_cov$AM_Cat))

AF_Cat <- as.numeric(as.character(check_cov$AF_Cat))

NR_Cat <- as.numeric(as.character(check_cov$NR_Cat))

R_Hyp <- as.numeric(as.character(check_cov$R_Hyp))

CharRace <- check_cov$CharRace

PatternNumber <- rep(NA, dim(data)[1])

PNID <- which(NB_Cat != "A" & NB_Cat != "B" &

!is.na(AM_Cat) & !is.na(AF_Cat) & !is.na(NR_Cat))

PatternNumber[PNID] <- NB_Cat[PNID] * 36 + AM_Cat[PNID] *

12 + AF_Cat[PNID] * 3 + NR_Cat[PNID] * 1 + 1

for (i in PNID) {

if (CharRace[i] != "??") {

Beta <- Wrk_Beta_all[data$Race[i], ]

LP1[i] <- NB_Cat[i] * Beta[1] + AM_Cat[i] * Beta[2] +

AF_Cat[i] * Beta[3] + NR_Cat[i] * Beta[4] + AF_Cat[i] *

NR_Cat[i] * Beta[6] + log(R_Hyp[i])

LP2[i] <- LP1[i] + NB_Cat[i] * Beta[5]

}

}

RR_Star1 <- exp(LP1)

RR_Star2 <- exp(LP2)

RR_Star <- cbind(RR_Star1, RR_Star2, PatternNumber)

RR_Star <- data.frame(RR_Star, row.names = NULL)

return(RR_Star)

}

absolute.risk <- function (data, Raw_Ind = 1, Avg_White = 0){

White_lambda1 <- c(1e-05, 7.6e-05, 0.000266, 0.000661, 0.001265,

0.001866, 0.002211, 0.002721, 0.003348, 0.003923, 0.004178,

0.004439, 0.004421, 0.004109)

White_lambda1Avg <- c(1.22e-05, 7.41e-05, 0.0002297, 0.0005649,

0.0011645, 0.0019525, 0.0026154, 0.0030279, 0.0036757,

0.0042029, 0.0047308, 0.0049425, 0.0047976, 0.0040106)

White_nlambda1 <- c(1.20469e-05, 7.46893e-05, 0.0002437767,

0.0005878291, 0.0012069622, 0.0019762053, 0.0026200977,

0.0033401788, 0.0039743676, 0.0044875763, 0.0048945499,

0.0051610641, 0.0048268456, 0.0040407389)

Black_lambda1 <- c(2.696e-05, 0.00011295, 0.00031094, 0.00067639,

0.00119444, 0.00187394, 0.00241504, 0.00291112, 0.00310127,

0.0036656, 0.00393132, 0.00408951, 0.00396793, 0.00363712)

Hspnc_lambda1 <- c(1.66e-05, 7.41e-05, 0.000274, 0.0006099,

0.0012225, 0.0019027, 0.0023142, 0.0028357, 0.0031144,

0.0030794, 0.0033344, 0.0035082, 0.0025308, 0.0020414)

Other_lambda1 <- c(1e-05, 7.6e-05, 0.000266, 0.000661, 0.001265,

0.001866, 0.002211, 0.002721, 0.003348, 0.003923, 0.004178,

0.004439, 0.004421, 0.004109)

FHspnc_lambda1 <- c(1.02e-05, 5.31e-05, 0.0001578, 0.0003602,

0.0007617, 0.0011599, 0.0014111, 0.0017245, 0.0020619,

0.0023603, 0.0025575, 0.0028227, 0.0028295, 0.0025868)

Chnes_lambda1 <- c(4.059636e-06, 4.5944465e-05, 0.000188279352,

0.000492930493, 0.000913603501, 0.001471537353, 0.001421275482,

0.001970946494, 0.001674745804, 0.001821581075, 0.001834477198,

0.001919911972, 0.002233371071, 0.002247315779)

Japns_lambda1 <- c(1e-12, 9.9483924e-05, 0.000287041681,

0.000545285759, 0.001152211095, 0.001859245108, 0.002606291272,

0.003221751682, 0.004006961859, 0.003521715275, 0.003593038294,

0.003589303081, 0.003538507159, 0.002051572909)

Filip_lambda1 <- c(7.500161e-06, 8.1073945e-05, 0.000227492565,

0.000549786433, 0.001129400541, 0.001813873795, 0.002223665639,

0.002680309266, 0.00289121923, 0.002534421279, 0.002457159409,

0.00228661692, 0.001814802825, 0.00175087913)

Hawai_lambda1 <- c(4.5080582e-05, 9.8570724e-05, 0.00033997086,

0.000852591429, 0.001668562761, 0.002552703284, 0.003321774046,

0.005373001776, 0.005237808549, 0.005581732512, 0.005677419355,

0.006513409962, 0.003889457523, 0.002949061662)

OtrPI_lambda1 <- c(1e-12, 7.1525212e-05, 0.000288799028,

0.000602250698, 0.000755579402, 0.000766406354, 0.001893124938,

0.002365580107, 0.00284393307, 0.002920921732, 0.002330395655,

0.002036291235, 0.001482683983, 0.001012248203)

OtrAs_lambda1 <- c(1.2355409e-05, 5.9526456e-05, 0.000184320831,

0.000454677273, 0.000791265338, 0.001048462801, 0.001372467817,

0.001495473711, 0.001646746198, 0.001478363563, 0.001216010125,

0.0010676637, 0.001376104012, 0.000661576644)

White_lambda2 <- c(0.000493, 0.000531, 0.000625, 0.000825,

0.001307, 0.002181, 0.003655, 0.005852, 0.009439, 0.015028,

0.023839, 0.038832, 0.066828, 0.144908)

White_lambda2Avg <- c(0.0004412, 0.0005254, 0.0006746, 0.0009092,

0.0012534, 0.001957, 0.0032984, 0.0054622, 0.0091035,

0.0141854, 0.0225935, 0.0361146, 0.0613626, 0.1420663)

White_nlambda2 <- c(0.0004000377, 0.0004280396, 0.0005656742,

0.0008474486, 0.0012752947, 0.0018601059, 0.0028780622,

0.0046903348, 0.0078835252, 0.0127434461, 0.0208586233,

0.0335901145, 0.0575791439, 0.1377327125)

Black_lambda2 <- c(0.00074354, 0.00101698, 0.00145937, 0.00215933,

0.00315077, 0.00448779, 0.00632281, 0.00963037, 0.01471818,

0.02116304, 0.03266035, 0.04564087, 0.06835185, 0.13271262)

Hspnc_lambda2 <- c(0.0003561, 0.0004038, 0.0005281, 0.0008875,

0.0013987, 0.0020769, 0.0030912, 0.004696, 0.007605,

0.0120555, 0.0193805, 0.0288386, 0.0429634, 0.0740349)

Other_lambda2 <- c(0.000493, 0.000531, 0.000625, 0.000825,

0.001307, 0.002181, 0.003655, 0.005852, 0.009439, 0.015028,

0.023839, 0.038832, 0.066828, 0.144908)

FHspnc_lambda2 <- c(0.0003129, 0.0002908, 0.0003515, 0.0004943,

0.0007807, 0.001284, 0.0020325, 0.0034533, 0.0058674,

0.0096888, 0.0154429, 0.0254675, 0.0448037, 0.1125678)

Chnes_lambda2 <- c(0.000210649076, 0.000192644865, 0.000244435215,

0.000317895949, 0.000473261994, 0.00080027138, 0.001217480226,

0.002099836508, 0.003436889186, 0.006097405623, 0.010664526765,

0.020148678452, 0.03799079659, 0.098333900733)

Japns_lambda2 <- c(0.000173593803, 0.000295805882, 0.000228322534,

0.000363242389, 0.000590633044, 0.001086079485, 0.001859999966,

0.003216600974, 0.004719402141, 0.008535331402, 0.012433511681,

0.020230197885, 0.037725498348, 0.106149118663)

Filip_lambda2 <- c(0.000229120979, 0.000262988494, 0.00031484409,

0.000394471908, 0.00064762261, 0.001170202327, 0.001809380379,

0.002614170568, 0.004483330681, 0.007393665092, 0.012233059675,

0.021127058106, 0.037936954809, 0.085138518334)

Hawai_lambda2 <- c(0.000563507269, 0.000369640217, 0.001019912579,

0.001234013911, 0.002098344078, 0.002982934175, 0.005402445702,

0.009591474245, 0.016315472607, 0.020152229069, 0.02735483871,

0.050446998723, 0.072262026612, 0.145844504021)

OtrPI_lambda2 <- c(0.000465500812, 0.00060046692, 0.000851057138,

0.001478265376, 0.001931486788, 0.003866623959, 0.004924932309,

0.008177071806, 0.00863820289, 0.018974658371, 0.029257567105,

0.038408980974, 0.052869579345, 0.074745721133)

OtrAs_lambda2 <- c(0.000212632332, 0.000242170741, 0.000301552711,

0.000369053354, 0.000543002943, 0.000893862331, 0.001515172239,

0.002574669551, 0.004324370426, 0.007419621918, 0.01325176513,

0.02229142749, 0.041746550635, 0.087485802065)

#***EDITED (START)***#

SG_lambda1 <- lambda[,"BC_INCIDENCE"]/100000

SG_CN_lambda1 <- lambda[,"BC_INCIDENCE_CHINESE"] /100000

SG_MY_lambda1 <- lambda[,"BC_INCIDENCE_MALAY"] /100000

SG_IN_lambda1 <- lambda[,"BC_INCIDENCE_INDIAN"]/100000

SG_lambda2 <- lambda[,"DEATH_INCIDENCE"]/1000

#***EDITED (END)***#

White_1_AR <- c(0.5788413, 0.5788413)

Black_1_AR <- c(0.7294988, 0.74397137)

Hspnc_1_AR <- c(0.749294788397, 0.778215491668)

Other_1_AR <- c(0.5788413, 0.5788413)

FHspnc_1_AR <- c(0.428864989813, 0.450352338746)

Asian_1_AR <- c(0.47519806426735, 0.50316401683903)

Avg_lambda1 <- array(0, dim = c(14, 5))

Avg_lambda1[, 1:(dim(Avg_lambda1)[2])] <- White_lambda1Avg

Avg_lambda2 <- array(0, dim = c(14, 5))

Avg_lambda2[, 1:(dim(Avg_lambda2)[2])] <- White_lambda2Avg

Wrk_lambda1_all <- rbind(White_lambda1, Black_lambda1, Hspnc_lambda1,

Other_lambda1, FHspnc_lambda1, Chnes_lambda1, Japns_lambda1,

Filip_lambda1, Hawai_lambda1, OtrPI_lambda1, OtrAs_lambda1,

SG_lambda1,SG_CN_lambda1,SG_MY_lambda1,SG_IN_lambda1) ## EDITED

Wrk_lambda2_all <- rbind(White_lambda2, Black_lambda2, Hspnc_lambda2,

Other_lambda2, FHspnc_lambda2, Chnes_lambda2, Japns_lambda2,

Filip_lambda2, Hawai_lambda2, OtrPI_lambda2, OtrAs_lambda2,

SG_lambda2,SG_lambda2,SG_lambda2,SG_lambda2) ## EDITED

Wrk_1_AR_all <- rbind(White_1_AR, Black_1_AR, Hspnc_1_AR,

Other_1_AR, FHspnc_1_AR, Asian_1_AR, Asian_1_AR, Asian_1_AR,

Asian_1_AR, Asian_1_AR, Asian_1_AR,

Asian_1_AR,Asian_1_AR,Asian_1_AR,Asian_1_AR) ## EDITED

AbsRisk <- rep(NA, dim(data)[1])

RR_Star <- relative.risk(data, Raw_Ind)

check_cov <- recode.check(data, Raw_Ind)

Error_Ind <- check_cov$Error_Ind

IDwoERR <- which(Error_Ind == 0)

for (i in IDwoERR) {

obs <- data[i, ]

rrstar1 <- RR_Star$RR_Star1[i]

rrstar2 <- RR_Star$RR_Star2[i]

One_AR_RR <- rep(NA, 70)

Strt_Intvl <- floor(obs$T1) - 20 + 1

End_Intvl <- ceiling(obs$T2) - 20 + 0

NumbrIntvl <- ceiling(obs$T2) - floor(obs$T1)

RskWrk <- 0

Cum_lambda <- 0

lambda1.temp <- array(0, dim = c(14, 5))

lambda2.temp <- array(0, dim = c(14, 5))

#***EDITED (START)***#

if(Avg_White==2){

One_AR1 <- Wrk_1_AR_all[obs$Race, 1]

One_AR2 <- Wrk_1_AR_all[obs$Race, 2]

One_AR_RR1 <- One_AR1 * rrstar1

One_AR_RR2 <- One_AR2 * rrstar2

One_AR_RR[1:30] <- One_AR_RR1

One_AR_RR[31:70] <- One_AR_RR2

lambda1.temp[, 1:(dim(lambda1.temp)[2])] <- Wrk_lambda1_all[obs$Race,

]

lambda2.temp[, 1:(dim(lambda2.temp)[2])] <- Wrk_lambda2_all[obs$Race,

]

lambda1 <- c(t(lambda1.temp))

lambda2 <- c(t(lambda2.temp))

}

#***EDITED (END)***#

if (Avg_White == 0) {

One_AR1 <- Wrk_1_AR_all[obs$Race, 1]

One_AR2 <- Wrk_1_AR_all[obs$Race, 2]

One_AR_RR1 <- One_AR1 * rrstar1

One_AR_RR2 <- One_AR2 * rrstar2

One_AR_RR[1:30] <- One_AR_RR1

One_AR_RR[31:70] <- One_AR_RR2

lambda1.temp[, 1:(dim(lambda1.temp)[2])] <- Wrk_lambda1_all[obs$Race,

]

lambda2.temp[, 1:(dim(lambda2.temp)[2])] <- Wrk_lambda2_all[obs$Race,

]

lambda1 <- c(t(lambda1.temp))

lambda2 <- c(t(lambda2.temp))

}

if (Avg_White == 1) {

One_AR_RR <- rep(1, 70)

lambda1.temp[, 1:(dim(lambda1.temp)[2])] <- Wrk_lambda1_all[obs$Race,

]

lambda2.temp[, 1:(dim(lambda2.temp)[2])] <- Wrk_lambda2_all[obs$Race,

]

if (obs$Race == 1 | obs$Race == 4) {

lambda1.temp <- Avg_lambda1

lambda2.temp <- Avg_lambda2

}

lambda1 <- c(t(lambda1.temp))

lambda2 <- c(t(lambda2.temp))

}

for (j in 1:NumbrIntvl) {

j_intvl <- Strt_Intvl + j - 1

if (NumbrIntvl > 1 & j > 1 & j < NumbrIntvl) {

IntgrlLngth <- 1

}

if (NumbrIntvl > 1 & j == 1) {

IntgrlLngth <- 1 - (obs$T1 - floor(obs$T1))

}

if (NumbrIntvl > 1 & j == NumbrIntvl) {

z1 <- ifelse((obs$T2 > floor(obs$T2)), 1, 0)

z2 <- ifelse((obs$T2 == floor(obs$T2)), 1, 0)

IntgrlLngth <- (obs$T2 - floor(obs$T2)) * z1 +

z2

}

if (NumbrIntvl == 1) {

IntgrlLngth <- obs$T2 - obs$T1

}

lambdaj <- lambda1[j_intvl] * One_AR_RR[j_intvl] +

lambda2[j_intvl]

PI_j <- ((One_AR_RR[j_intvl] * lambda1[j_intvl]/lambdaj) *

exp(-Cum_lambda)) * (1 - exp(-lambdaj * IntgrlLngth))

RskWrk <- RskWrk + PI_j

Cum_lambda <- Cum_lambda + lambdaj * IntgrlLngth

}

AbsRisk[i] <- 100 * RskWrk

}

return(AbsRisk)

}

References

Gail, M. H., Brinton, L. A., Byar, D. P., Corle, D. K., Green, S. B., Schairer, C., & Mulvihill, J. J. (1989). Projecting individualized probabilities of developing breast cancer for white females who are being examined annually. *J Natl Cancer Inst, 81*(24), 1879-1886. doi:10.1093/jnci/81.24.1879

National Registry of Diseases Office, N. (2017). *Singapore Cancer Registry Annual Registry Report 2015*. Retrieved from <https://www.nrdo.gov.sg/docs/librariesprovider3/Publications-Cancer/cancer-registry-annual-report-2015_web.pdf%3Fsfvrsn%3D1dd97be4_10>

Statistics, D. o. (2017, 2020). M810141 - Age-Specific Death Rates, Annual Retrieved from <https://www.tablebuilder.singstat.gov.sg/publicfacing/createDataTable.action?refId=13249>
